# Thoracic stent-graft herniation through the aortic wall in a case of lung cancer

**DOI:** 10.1093/icvts/ivab277

**Published:** 2021-11-15

**Authors:** Naritomo Nishioka, Yoshihiko Kurimoto, Masaru Abe, Hiroaki Kato

**Affiliations:** 1 Department of Cardiovascular Surgery, Teine Keijinkai Hospital, Sapporo, Japan; 2 Department of Thoracic Surgery, Teine Keijinkai Hospital, Sapporo, Japan

**Keywords:** Thoracic endovascular aortic repair, Bleeding and migration, Lung cancer

## Abstract

A 67-year-old man had left upper lung cancer with invasion into the descending aorta. He underwent pre-emptive thoracic endovascular aortic repair using a Valiant Navion followed by left lung upper lobectomy with resection of the aortic wall. Because of continuous bleeding, he underwent re-thoracotomy. Since the surgically resected aortic wall was largely cleaved, bleeding around the stent-graft that herniated into the left pleural cavity was observed. Re-thoracic endovascular aortic repair using a GORE TAG was immediately performed to prevent further stent-graft herniation and impending lethal haemorrhage. It may be necessary to consider reinforcement of the resected aortic wall to prevent thoracic endovascular aortic repair-related complications.

## INTRODUCTION

A surgical procedure combined with resection of the lung and aortic wall for lung cancer is highly invasive and is a challenging strategy for thoracic surgeons. Resection of the aortic wall has been safely performed in recent years with a less invasive approach of thoracic endovascular aortic repair (TEVAR) [1]. Nevertheless, TEVAR-related complications in this strategy should be examined. Here, we report on bleeding and stent-graft migration secondary to cleaving a resected aortic wall in the case of simultaneous TEVAR combining lung lobectomy and resection of the aortic wall for advanced lung cancer as a pitfall of this strategy.

## CASE REPORT

**Figure 1:**
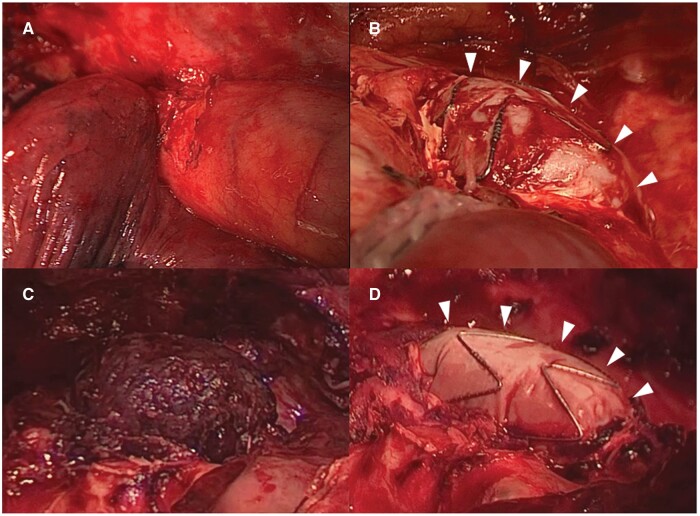
Intraoperative findings. (**A**) The upper lobe lung cancer infiltrated the descending aorta. (**B**) Exposure of the stent graft after resection of the aortic wall (arrowheads). (**C**) There was a large haematoma above the stent graft when the patient underwent re-thoracotomy. (**D**) Herniation of the stent graft was confirmed after resection of the large haematoma (arrowheads).

A 67-year-old man presented with bloody sputum. Computed tomography revealed a solid mass and the descending aorta invasion, and no lymphadenopathy or metastasis. Transbronchial biopsy revealed squamous cell carcinoma. The surgical treatment was planned to resect the left upper lobe and the cancer-invaded aortic wall (clinical stage IIIA: T4N0M0). Scheduled pre-emptive TEVAR followed by left lung upper lobectomy and resection of the cancer-invaded aortic wall was carried out. First, using a retrograde right common femoral approach, the Valiant Navion Thoracic Stent Graft System, Medtronic Vascular, Santa Rosa, CL was employed. The Valiant Navion with a physician-crafted fenestration to preserve the left subclavian artery was deployed from zone 2 to the descending aorta. The diameter of Valiant Navion FreeFlo was chosen to have a 36-mm taper, as the diameter of the zone 2 aortic arch ranged from 29 to 27 mm, resulting in 24% oversizing. The proximal and distal neck lengths of the aorta were over 40 mm to secure bleeding prevention during the planned aortic wall resection. Digital subtraction angiography revealed no endoleaks. Second, an *en bloc* tumour resection was performed that included resection of the left upper lobe and the cancer-invaded aortic wall (Fig. 1A and B). The aortic wall was slightly vulnerable due to the inflammatory changes around the tumour infiltration.

**Figure 2:**
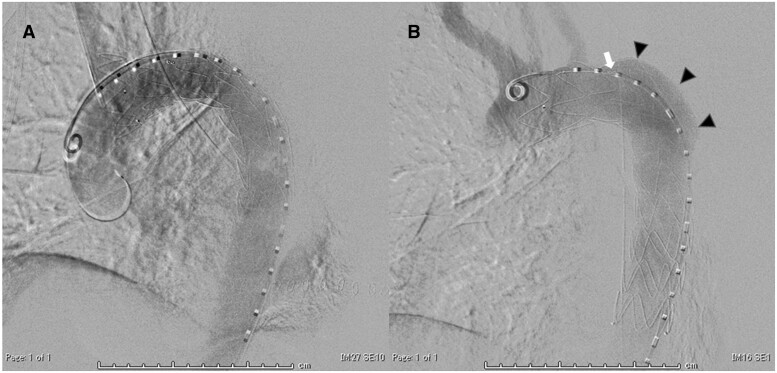
Digital subtraction angiography in thoracic endovascular aortic repair (TEVAR) and re-TEVAR. (**A**) After the first TEVAR. (**B**) Before re-TEVAR. Migration of the stent graft was confirmed compared to after the first TEVAR (arrowheads). The marker (white arrow) of the distal end of a fenestration also moved close to free space of the left chest cavity.

After the operation, the patient’s mean blood pressure was kept as high as 80 mmHg+ to prevent spinal cord ischaemia. Because of continuous bleeding until the day after the operation, he underwent re-thoracotomy. A haematoma was observed above the resection site of the descending aorta. After the haematoma was removed, bleeding and stent-graft herniation into the chest cavity caused by cleaving the resected aortic wall was observed (Fig. 1C and D). Bleeding was assumed to be type I and IV endoleaks. Digital subtraction angiography revealed visible migration (Fig. 2). Zone 2 re-TEVAR with a GORE TAG Conformable thoracic stent graft with ACTIVE CONTROL system (W.L. Gore & Associates, Inc., AZ, USA) (31 mm × 150 mm) was immediately performed to prevent further stent-graft migration and impending lethal haemorrhage, although the initially preserved left subclavian artery was intentionally covered. The patient had a haemodynamically stable postoperative course. He was discharged on the 28th postoperative day and was doing well at the 3-month postoperative follow-up.

## DISCUSSION

The surgical strategy for T4 lung cancer invading the aorta is challenging for thoracic surgeons. Previous study has described the *en bloc* resection of tumours that invaded the aorta using a cardiopulmonary bypass [2]. This surgical strategy was associated with significant mortality and morbidity. In contrast, aortic wall resection with TEVAR has been reported as a less invasive procedure [1]. Our strategy with simultaneous TEVAR and the *en bloc* resection of tumours was considered to be acceptable.

As for TEVAR with the zone 2 landing in this case, we chose a physician-crafted fenestration to preserve the left subclavian artery under the anatomical indication. We ensured a distance of at least 10 mm (generally, 15 mm) between the fenestration site and the proximal end of the aortic aneurysm or primary entry in the case of aortic dissection to ensure sufficient sealing of the device. This technique is feasible and good results have been reported [3]. We secured more than 30 mm in length as a proximal neck in this case, taking possible aortic wall resection into consideration. The reasons why haemothorax secondary to stent-graft migration occurred were examined. First, the aortic wall may have been vulnerable because of the inflammatory changes. Our impression was that the aortic wall was slightly soft. Reinforcement of the aorta through patch plasty with bovine pericardium should always be considered, regardless of the size and position of the defect [4]. This reinforcement is thought to be useful for not only preventing bleeding and migration but also decreasing the risk of infection and fistulization. Nevertheless, a simple endovascular strategy is attractive. We have rarely experienced stent-graft distal migration in degenerative aortic aneurysms when using Valiant Thoracic Stent Graft with the Captivia Delivery System, Medtronic Vascular, Santa Rosa, CL with a proximal bare stent. The present case obviously had a healthy aortic wall, which increased the risk of stent-graft migration. Considering the clinical course of this case, relining a second stent graft in the first one might be an option to prevent stent-graft herniation through the resected aortic wall. Second, the blood pressure was maintained slightly high to prevent spinal cord ischaemia after operation, which is a well-known complication after TEVAR. This higher blood pressure possibly contributed to the complications. Third, the resection site of the aorta was located on the greater curvature side. The aortic wall on the greater curvature side was haemodynamically influenced by blood flow [5]. It is highly possible that migration occurred along with the effects of blood pressure.

In conclusion, simultaneous TEVAR and *en bloc* resection of tumours can be considered feasible, although bleeding and migration from the resection site of the aorta are a pitfall that must be carefully monitored for. Clinicians may need to consider concomitant reinforcement for the aortic wall after the resection.

**Conflict** **of interest:** none declared.
